# Integrating mass spectrometry and hyperspectral imaging for protoporphyrin IX detection in malignant glioma tissue

**DOI:** 10.1038/s41598-025-26245-0

**Published:** 2025-11-04

**Authors:** Fabio D’Alessandro, Anna Walke, Nora Maren Kiolbassa, Walter Stummer, Simone König, Eric Suero Molina

**Affiliations:** 1 Department of Neurosurgery, University Hospital Münster, Albert-Schweitzer-Campus 1 A1, 48149 Münster, Germany; 2 Core Unit Proteomics, Interdisciplinary Centre for Clinical Research, University of Münster, Münster, Germany; 3 Macquarie Medical School, Macquarie University, Sydney, Australia

**Keywords:** Biological fluorescence, Optical spectroscopy, Biomarkers, Diagnostic markers

## Abstract

Maximal safe tumor resection is crucial for the treatment of high-grade gliomas (HGG). 5-aminolevulinic acid (5-ALA)-mediated fluorescence-guided surgery enhances tumor visualization by inducing protoporphyrin IX (PpIX) accumulation. However, current fluorescence-based observation devices lack the sensitivity for detecting tumor cells in low-density infiltrative zones. Hyperspectral imaging (HI) offers a potential solution. In this study, HI-derived PpIX measurements were compared to those obtained from reversed-phase liquid chromatography coupled to mass spectrometry (LC–MS), a method that delivers accurate concentrations. Additionally, we investigated coproporphyrins (Cp) I and III, since they potentially interfere with PpIX determination. Pig brain was used as a surrogate for protocol development and acquisition of comparative HI and LC–MS reference data, which were subsequently used to evaluate the results obtained from 27 biopsies from nine patients undergoing 5-ALA-mediated tumor resection. During sample preparation for LC–MS, 80% PpIX and 45% combined Cp I & III were recovered from brain tissue. For LC–MS quantification of PpIX, accuracy ranged from 98 to 137%, and coefficient of variation was 5–14%, indicating sufficient precision. For HI, the values were 77–121% and 11–31%, respectively. Notably, HI significantly overestimated PpIX concentrations compared to those determined by LC–MS. This study highlights LC–MS as a reliable method for porphyrin quantification and suggests that HI workflows need further optimization for accurate tumor delineation in HGG.

**Supplementary Information:**

The online version contains supplementary material available at 10.1038/s41598-025-26245-0.

## Introduction

The prognosis of high-grade glioma (HGG) patients correlates with the extent of tumor resection^1^. Excess administration of 5-aminolevulinic acid (5-ALA) leads to protoporphyrin IX (PpIX) accumulation in malignant gliomas, guiding surgical resection, as PpIX can be excited to fluoresce. This technique, known as fluorescence-guided surgery (FGS), maximizes tumor resection^2^. 5-ALA is administered orally to the patient before surgery and converted to PpIX as part of the natural heme biosynthesis, subsequently accumulating in HGG tissue. The exact mechanisms are still unclear, but factors such as the disruption of the blood–brain barrier, as commonly observed in HGG^3,4^, altered metabolism of tumor cells, and the hypoxic tumor environment^5^ appear to play a role. Moreover, iron deficiency and decreased ferrochelatase (FECH) activity may also contribute to PpIX accumulation^6^.

Porphyrin synthesis starts with the formation of 5-ALA^7,8^, catalyzed by 5-ALA-synthase inside the mitochondria, which is considered the rate-limiting step and is subject to negative feedback regulation by heme^9^—that is bypassed in case of a 5-ALA excess, as in FGS. In heme synthesis, intermediates such as uroporphyrin III (Up III), coproporphyrin III (Cp III), and PpIX are formed. The end product, heme, is ultimately synthesized from PpIX by the insertion of a divalent iron ion, catalyzed by FECH^10,11^. Up III and Cp III are generated as their type-I isomers by spontaneous cyclization followed by decarboxylation. Up I / III and Cp I / III have been described to accumulate with PpIX within necrotic tissue of squamous cell carcinoma after 5-ALA administration^11,12^.

Certain commercially available surgical microscopes used in FGS can switch from conventional white light illumination to violet-blue light to excite PpIX. PpIX fluorescence is visualized using a dedicated combination of excitation (transmission maximum ~ 400 nm) and long-pass emission filters, transmitting broadband light > 450 nm of the electromagnetic spectrum^13^. PpIX fluorescence intensity is somewhat related to tumor cell density^14,15^ and thus useful to locate tumor tissue. However, 5-ALA-mediated fluorescence-guidance can detect tumor tissue only to a limited extent^16^. With the diffusely infiltrative growth pattern of gliomas, this limitation significantly contributes to the near inevitability of tumor recurrence^17^. About 85% of tumors recur in the vicinity of the resection cavity^17^, implicating the diffuse infiltrative nature of malignant glioma and the poor tumor tissue identification at the border zones.

Hyperspectral imaging (HI) enhances the sensitivity and specificity of PpIX detection compared to surgical microscopes, enabling PpIX measurement even in non-visibly fluorescing tumor samples^18,19^. However, spectral imaging systems are still under development and limited to research use. HI devices measure spatially resolved spectral data over a pre-defined wavelength range of the electromagnetic spectrum^18,20^. Signal processing of HI data involves normalization to correct for tissue optical properties, primarily absorption and scattering^21^. Separating signals from different sources is achieved by spectral unmixing using known basis spectra of endogenous fluorophores such as PpIX, flavin, lipofuscin, NADH, a.o.^22,23^.

The local microenvironment influences PpIX fluorescence. Factors such as pH impact PpIX fluorescence emission and aggregation behaviour^24–27^. In addition, two different physicochemical states have been described for PpIX, further complicating its quantification^22^. Moreover, different porphyrins potentially accumulate in the exact location as PpIX, which affects the measured fluorescence intensity. Because porphyrins share a common core structure, all members of this substance class have overlapping absorption and fluorescence emission spectra^28^. Thus, distinguishing porphyrins remains challenging when relying solely on optical methods^29–32^.

Fluorophore quantification by spectral imaging requires careful consideration of method-inherent processes, including an appropriate calibration strategy. Currently, for calibration of HI devices, so-called phantoms (mixtures of a dye, intralipid, and PpIX) are used as reference compounds, which mimic the optical properties of potential analytes such as brain tissue^19,33,34^. However, these phantoms do not sufficiently reflect actual tissue properties, such as pH, consistency, composition, and the presence of unknown fluorophores that cause autofluorescence^18^. Therefore, we introduce pig brain as a surrogate matrix for analytical purposes. We used a mass spectrometry (MS)-based approach for PpIX quantification in brain tissue, complementing HI, as MS enables the specific and accurate detection of individual porphyrins in biological samples. We extended our earlier MS method for PpIX measurement in serum^10^, which has already been used in a clinical study demonstrating that PpIX can potentially serve as a blood biomarker for HGG^35^. We applied the method to malignant glioma tissue in the present study and extended it to target Cp I and Cp III next to PpIX. For reference purposes, we determined the concentration of these different porphyrins in spiked pig brain surrogate tissue using both MS and HI to evaluate the results of optical PpIX detection. Finally, a cohort of 27 human HGG biopsies was investigated using MS and HI techniques for direct comparison.

## Methods

### Permissions

The study was conducted in accordance with the ethical standards outlined in the Declaration of Helsinki and with approval from the local Ethics Committee of the University of Münster and the Ärztekammer Westfalen-Lippe (approval number: 2023-492-f-S). All patients provided informed written consent. Experiments with pig brains were permitted by the Health and Veterinary Office Münster (Reg.-No. 05 515 1052 21).

### Chemicals

PpIX (Enzo Life Sciences GmbH, Lörrach, Germany), mesoporphyrin (MpIX, Merck KGaA, Darmstadt, Germany), Cp I and Cp III (Frontier Specialty Chemicals, Logan, Utah, USA) were dissolved in dimethyl sulfoxide (DMSO, Merck KGaA, Darmstadt, Germany) yielding stock solutions of 300 pmol/µl.

### Pig brain surrogate tissue

Pig brain was obtained from a local butcher. Only cerebrum was used for this study; it was homogenized using a blender (VDI 12, VWR International, Hannover, Germany) and stored at -20 °C until use. For liquid chromatography (LC) coupled to MS analysis, 250 mg pig brain homogenate was spiked with PpIX, resulting in concentrations of 0.002, 0.01, 0.04, 1.0, 2.5, and 3.0 pmol/mg. For spectral imaging, 600 mg pig brain homogenate was spiked with PpIX stock solution (300 pmol/µl) to the desired concentration (see Sect. 3.4) and shaken at room temperature for 30 min prior to porphyrin extraction or HI measurement. Measurements of spiked pig brain with the HI system and LC–MS were conducted with three technical replicates.

### HI data acquisition and preprocessing

HI measurements were performed ex vivo using the previously described wide-field imaging device^18,22,25^*.* The hyperspectral camera was built using an OPMI pico microscope (CZM, Oberkochen, Germany). It harbored a 405 nm light-emitting diode (LED) for excitation, a scientific complementary metal oxide semiconductor (sCMOS) camera (PCO edge 4.2, PCO, Kehlheim, Germany), a liquid crystal tunable filter (LCTF, Meadowlark Optics Inc., Colorado, USA) and a color camera (IDS Imaging Development Systems GmbH, Obersulm, Germany) with an IMX252 sensor (Sony, Tokyo, Japan). Measurements were performed in fluorescence (405 nm), broadband white light (400–750 nm), and dark mode with the light source turned off. Images were taken wavelength-dependently by switching the LCTF from 420 to 730 nm in 3 nm steps for fluorescence and dark mode and 5 nm steps, respectively, for broadband white light measurement. Residual light was recorded with the IDS camera, resulting in a color image. In front of the camera, a filter wheel equipped with a BLUE 400 filter (Carl Zeiss Meditec AG, Oberkochen, Germany) was installed to capture a BLUE 400 image in addition to the hyperspectral measurement.

MATLAB (The MathWorks Inc., Natick, Massachusetts) was used to analyze HI spectra^18^. The HI analysis algorithm determines the PpIX contribution in units of µg/ml^22^. For comparison of HI data with LC–MS results during clinical HGG biopsy evaluation, spiked pig brain homogenates at PpIX concentrations of 0 (native), 0.002, 0.01, 0.04, 0.2, 1.0, 2.0, 2.5, 3.0, 5.0, 7.0, 9.4, 12.0, 14.0, 20.0, 50.0 pmol/mg were measured with the HI camera. The results from these measurements allowed the conversion of the hyperspectrally measured PpIX contribution in µg/ml (HI_PpIX_) to a value in pmol/mg (HI_Calc-PpIX_).

### MS-based porphyrin analyses

LC–MS measurements were performed using an HP1100 HPLC (Agilent, Waldbronn, Germany) coupled to an Esquire 3000 ion trap mass spectrometer (Bruker Corp., Bremen, Germany). MpIX was chosen as an internal standard due to its high structural similarity to PpIX. Reversed-phase LC–MS was performed as described before^10^ with few modifications to accommodate Cp I/III analysis (for details on instrument parameters, see Supplemental Digital Content 1, Table S1). Cp I / III, MpIX, and PpIX were quantified using the total areas for the three most abundant fragment ions in MS/MS mode (Table 1) using Skyline software (version 20.1^36^). Raw data were converted with the msConvert toolkit from ProteoWizard software (version 3^37^).

**Table 1:** Parameter for time-dependent MS/MS fragmentation of porphyrins. Isolation time and m/z window, and theoretical masses for the expected fragment ions used for quantification (for more details on instrument parameters, see Supplementary Table S1).

Analyte	Time [min]	Isolation *m*/*z* ± 2.0	Fragmentation amplitude [V]	Fragment ions for quantification *m*/*z*
Cp I	8.8–9.0	655.3	0.62	596.3; 537.3; 523.3
Cp III	9.0–9.2	655.3	0.62	596.3; 537.3; 523.3
MpIX	13.1–13.3	567.3	0.90	493.3; 479.3; 449.3
PpIX	14.8–15.1	563.3	0.95	489.3; 459.3; 445.3

For quantification of porphyrins in clinical HGG biopsies, pig brain homogenates spiked with PpIX, Cp I, and Cp III in concentrations each of 0 (native level), 0.01, 0.04, 0.2, 1.0, 2.0, 2.5, 3.0, 5.0 and 7.0 pmol/mg were analyzed with LC–MS (LC-MS_PpIX_). Due to the low native content of Cp I and Cp III, they were combined to form total LC-MS_Cp_. Matrix effects were calculated by comparing peak areas of porphyrins spiked to the reconstituted residue from brain tissue extraction with results for the pure substances measured in DMSO.

### Porphyrin extraction from brain tissue

Before tissue extraction, MpIX was added at 2 pmol/mg. Porphyrins were extracted from brain tissue of up to 500 mg by adding 1 ml extraction buffer, which consisted of one part 1 M HCl mixed with two parts of a solution containing 80% acetonitrile (ACN) and 20% DMSO as described previously^38^ using an ultrasonic rod (Labsonic M, Sartorius Lab Instruments GmbH & Co. KG, Göttingen, Germany). Samples were centrifuged (20 min, 10.000 rcf, 18 °C) and the supernatant was collected. The extraction process was repeated once, and supernatants were pooled and diluted with 4 ml water to achieve a maximum organic content of 7%.

Porphyrins were purified by solid phase extraction (SPE; OASIS HLB 3 cc, 30 mg, Waters GmbH, Eschborn, Germany). The diluted supernatant (~ 6 ml) was loaded onto the SPE cartridge and washed with 80% methanol (MeOH, 10 ml). Porphyrins were eluted with 2 ml of 2% formic acid (FA) in ACN, and eluates were dried using a vacuum concentrator (SpeedVac, Savant SPD 111 V with vapor trap Savant RVT 5105, Thermo Fisher Scientific, Schwerte, Germany). The residue was reconstituted in DMSO at a volume (in µl) twice that of the initial tissue mass (in mg). The DMSO volume for reconstitution was at least 40 µl, as this was the minimal volume required for LC–MS analysis with three technical replicates. Prior to LC–MS, samples were filtered using wwPTFE centrifugal filter units (Pall GmbH, Dreieich, Germany).

For this protocol, porphyrin recovery and the matrix effect were tested. For the latter, pig brain homogenates were extracted, but porphyrins were spiked at the end of the procedure during reconstitution with DMSO.

### Patients

For this proof-of-concept study, 27 tumor biopsies were analyzed from nine patients (1–5 biopsies per patient) undergoing surgery for resection of lesions suspicious of HGG (Table 2). Biopsies ranged from 1.6 to 189.9 mg tissue with a median weight of 18.2 mg. Patients received 5-ALA (Gliolan, medac, Wedel, Germany) at a dose of 20 mg/kg body weight four hours before induction of anesthesia. Tumor entities were classified according to the 2021 WHO classification of CNS tumors^39^. Before biopsy collection, the visual fluorescence was graded into the categories “none” and “strong” by the surgeon using a KINEVO 900 surgical microscope equipped with a BLUE 400 filter system (Carl Zeiss Meditec (CZM) AG, Oberkochen, Germany). Tumor samples were immediately analyzed after FGS using the hyperspectral microscopic device, as described in Section *HI data acquisition and preprocessing* above. Subsequently, samples were stored at −20 °C until extraction for LC–MS analysis.

**Table 2:** Patient characteristics for porphyrin analysis in malignant glioma tissue.

		Patients	Biopsies
Gender	Male	8	25
	Female	1	2
Age		51 ± 12 Range 24–62	
WHO grade	3	1	4
	4	8	23
Fluorescence	None	4*	10
	Strong	7*	17
Primary / Recurrent tumor	Primary	3	11
	1st recurrence	3	8
	2nd recurrence	3	8
Histology	Oligodendroglioma (WHO 3)	1	4
	Glioblastoma	8	23

*Both fluorescing and non-fluorescing samples were collected from two patients.

## Results

### LC–MS analysis of coproporphyrins – method development

**Fig. 1:**
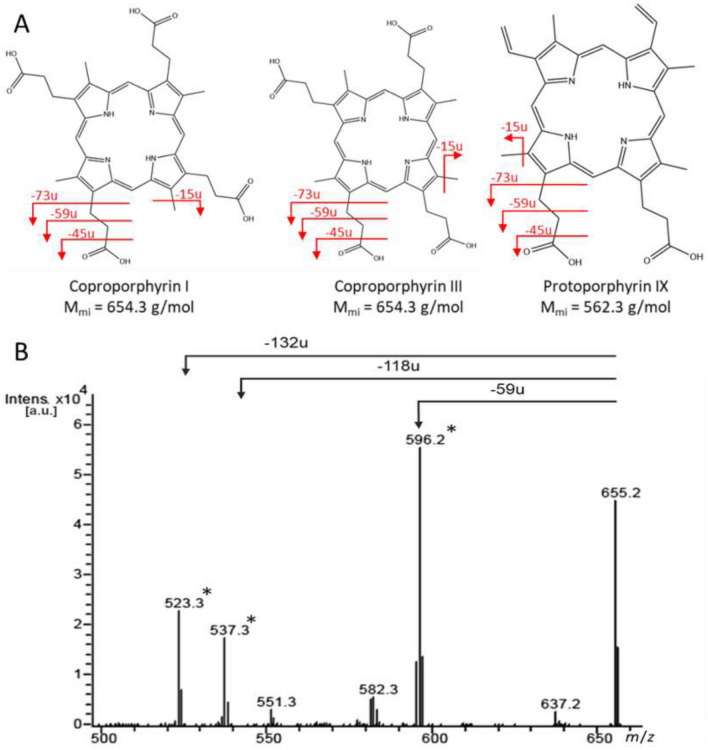
(**A**) Structures of type I and III isomers of coproporphyrin and their corresponding monoisotopic mass compared to PpIX. Major MS/MS fragmentation sites are labeled in red; fragmentation preferentially occurs on the sidechains of the tetrapyrrole core structure. (**B**) MS/MS spectrum of Cp III measured with ion trap MS. The MS/MS spectrum of Cp I looks the same due to the isomeric structure of the molecules. Ions labeled with an asterisk (*) were used for specific detection and quantification. This figure is original to this submission, so no credit or license is needed.

**Fig. 2:**
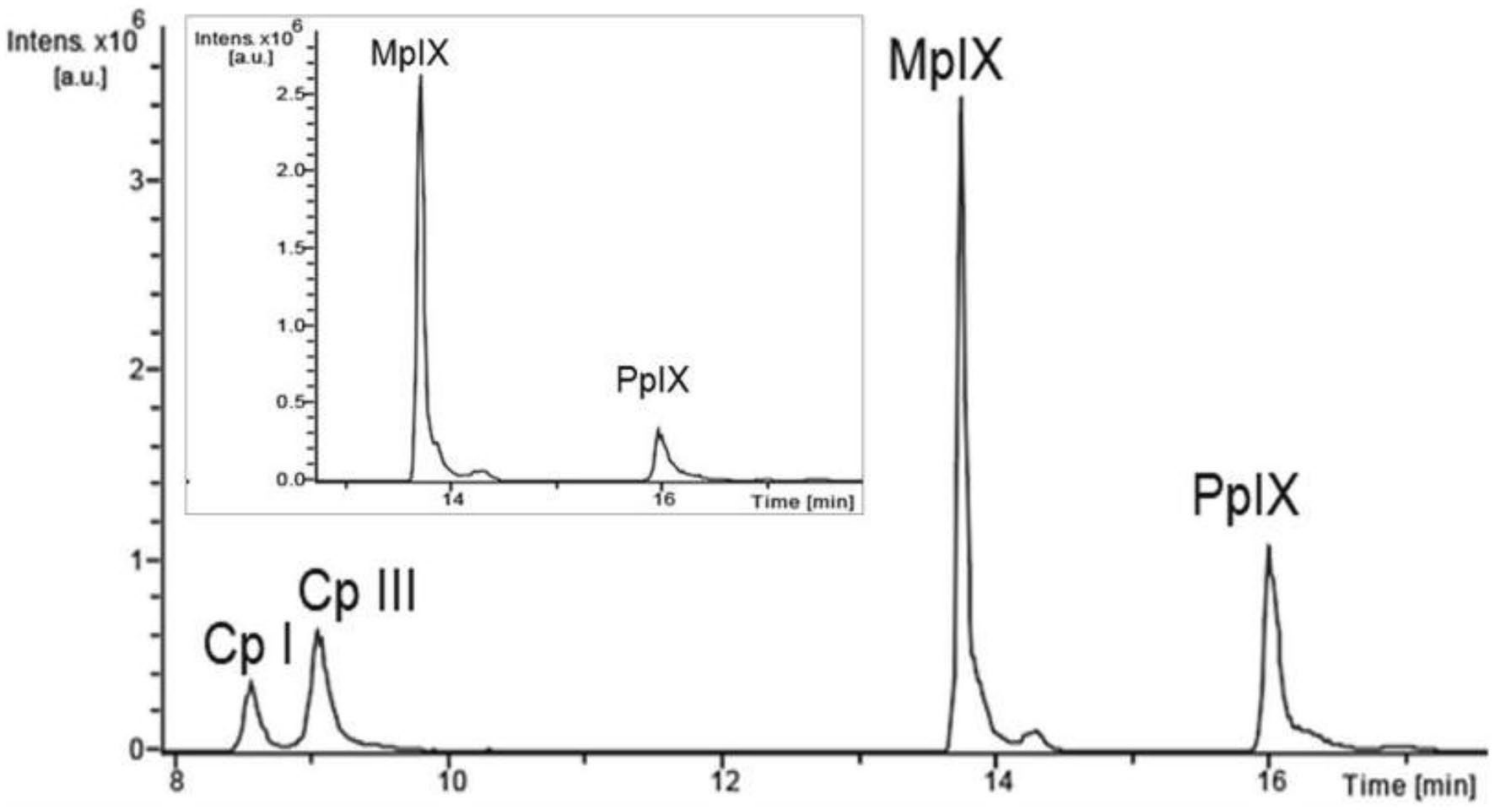
LC chromatogram of a porphyrin standard in DMSO and an LC trace obtained from a non-fluorescing biopsy (inset). The Cp concentration in the biopsy was comparatively low (intensity < 6.5*10^4^), so that only MpIX and PpIX are shown. This figure is original to this submission, so no credit or license is needed.

Mass spectra and gas-phase fragmentation of PpIX and MpIX have been described in previous work^10^. Cp I / III structures differ from these porphyrins in the side chains (Fig. 1A). Cp I / III ionize as singly-charged [M + H]⁺ ion species at the same mass-to-charge ratio (*m*/*z* 655.3) in positive electrospray ionization (ESI) mode. MS/MS fragmentation preferentially occurs on the side chains of the tetrapyrrole core structure in the same manner as noted for PpIX and MpIX^10^. The most abundant fragment ion is generated by the loss of an ethanoic acid substituent (-CH_2_COOH, 59 u, Fig. 1B). Further fragment ions result from side chain losses of 118 u (2 × 59 u; or 73 u and 3 × 15 u for methyl groups) and 132 u (59 u and propionic acid substituent (-CH_2_CH_2_COOH, 73 u)) as described before^40^. These losses generated the most intense fragment ions in MS/MS and were thus used for molecule-specific detection. For Cp I/III, due to their isomeric structure, MS analysis alone would not have been sufficient for unambiguous identification; however, the species were well separated by reversed-phase LC (Fig. 2). No carry-over of individual porphyrins was detected with the chosen LC column and gradient.

### Pig brain analysis

#### Porphyrin extraction and LC–MS

The extraction of porphyrins from brain tissue was achieved with 80 ± 2% (average (av) ± standard deviation (sd)) recovery for PpIX and about 45 ± 1% for Cp I and Cp III, respectively. For PpIX, recovery rates were superior to other studies extracting porphyrins from liver (50%^38^) or oral bacteria (55%^41^). For Cp, recovery was slightly lower compared to the same studies (50% for liver, 99% for Cp I for oral bacteria) due to the SPE washing step with 80% MeOH, which, however, is needed for best PpIX recovery.

Regarding the matrix effect, Cp I was determined at 102 ± 2% in tissue compared to the reference, Cp III at 100 ± 1%, MpIX at 94 ± 2%, and PpIX at 178 ± 2%. For Cp I, Cp III, and MpIX, similar matrix effects were described by Fyrestam et al. (Cp I 113%; Cp III 99%; MpIX 96%)^41^, but not for PpIX (86%). The increased matrix effect in pig brain tissue compared to oral bacteria matrix (Fyrestam^41^) is due to a native PpIX content above the limit-of-detection (LOD), which was confirmed by regular LC–MS measurements of blank matrix.

Porphyrin quantification was possible with an on-column LOD of 3 fmol (≙ 0.6 fmol/mg) for PpIX and 2 fmol (≙ 0.4 fmol/mg) for Cp I / III. Limit-of-quantification (LOQ) was 20 fmol on-column (≙ 4 fmol/mg) for PpIX and 10 fmol (≙ 2 fmol/mg) for Cp I / III. The LOQ agreed with previous studies (Cp I: 7.3 fmol, PpIX: 2.7 fmol on-column^41^).

Data were linear within the 0.01 to 7.0 pmol/mg range for PpIX. Due to the low concentrations of Cp I and Cp III in tissue, we summed up their concentrations to “total Cp” (denoted Cp) for further consideration. For the same reason, Cp was analyzed in the narrower range of 0.02 to 5.0 pmol/mg (for data curves, see Supplemental Digital Content 2, Fig. S1).

Precision was determined by measuring the identical sample three times and analyzing the degree of random variation. Within the tested range, precision ranged from 5 to 14% except for the lowest concentration, which had a precision of 30% for PpIX and 3–11% for Cp.

Measurement accuracy for PpIX detection was tested in pig brain homogenate by spiking three samples with a known concentration of 3.0 pmol/mg PpIX and subsequently using the developed protocol for PpIX quantification. Thereby, an accuracy of 100 ± 6% was achieved. All data regarding LC–MS method validation can be found in the Supplement section (see Supplemental Digital Content 3–6, Tables S2–S5).

#### MS and HI analyses of spiked pig brain surrogate

**Fig. 3:**
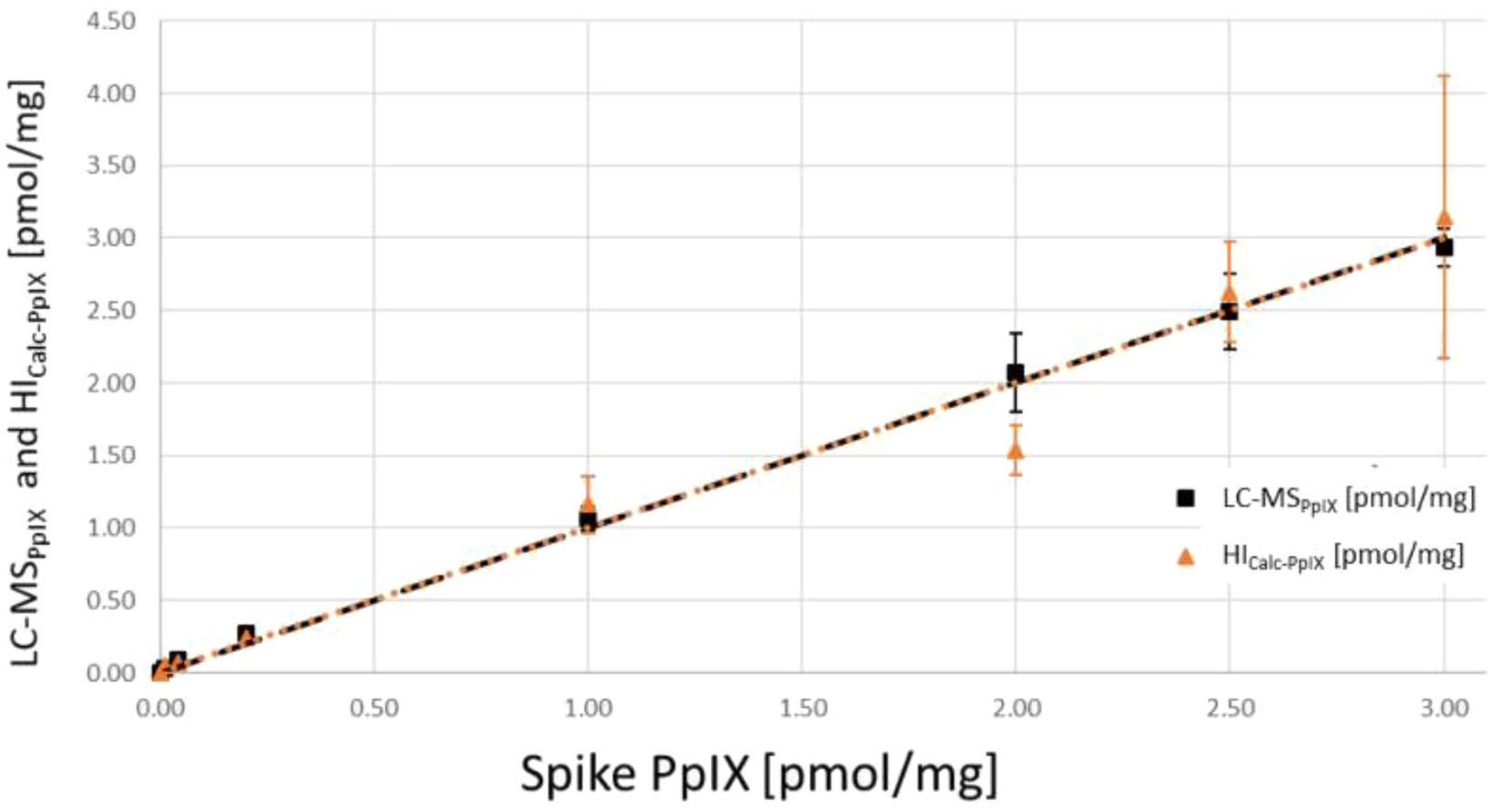
Results for the PpIX analysis of spiked pig brain tissue. LC-MS_PpIX_ (black squares) and HI_Calc-PpIX_ (orange triangles) in pmol/mg for direct comparison of both techniques (x-axis: spiked concentration, y-axis: measured values). This figure is original to this submission, so no credit or license is needed.

The results of the reference experiments in pig brain tissue homogenate are given in Table 3. Data were linear in both cases (see Fig. 3 and Supplemental Digital Content 7, Fig. S2, for data plots). At minimal spiked PpIX levels close to the LOQ (0.01 and 0.04 pmol/mg), the error in both systems was substantial, with accuracies of up to 506% for HI and 346% for LC–MS. Above 0.04 pmol/mg spiked PpIX, LC–MS had an accuracy of 98–137% and a precision ranging from 5–14%. Accuracy and precision of HI varied between 77 and 121% and 11–31%, respectively. When measuring higher PpIX concentrations of up to 50 pmol/mg with HI, which was required for the evaluation of some clinical biopsies, the values for accuracy and precision were improved for samples above a spiked PpIX concentration of 7.0 pmol/mg to 82–104% and 2–18%, respectively, using a square instead of a linear fit (see Supplemental Digital Content 8, Fig. S3).

**Table 3:** Results for LC–MS and HI analyses of pig cerebrum homogenate spiked with PpIX.

Spike PpIX [pmol/mg]	HI_PpIX_ [µg/ml]	HI_Calc-PpIX_ [pmol/mg]	LC-MS_PpIX_ [pmol/mg]
0.01	0.01 ± 0.00	0.05 ± 0.02	0.04 ± 0.01
0.04	0.01 ± 0.00	0.07 ± 0.03	0.09 ± 0.01
0.20	0.04 ± 0.00	0.24 ± 0.03	0.27 ± 0.04
1.00	0.19 ± 0.03	1.16 ± 0.20	1.06 ± 0.08
2.00	0.25 ± 0.03	1.54 ± 0.17	2.07 ± 0.27
2.50	0.43 ± 0.06	2.62 ± 0.35	2.49 ± 0.26
3.00	0.52 ± 0.16	3.15 ± 0.98	2.93 ± 0.13

Average and standard deviation values of three technical replicates.

In summary, within the examined concentration range, LC–MS enabled correct PpIX determination. Accuracy and precision of HI were also good, promising acceptable PpIX measurement in unknown samples. Our experiments provide a reference data set for the used camera setup and algorithm to routinely convert the typical HI_PpIX_ output given in µg/ml to HI_Calc-PpIX_ in pmol/mg. These results enable the comparison of PpIX results determined in biopsies with those obtained by LC–MS and HI. It is, however, essential to remember that PpIX was evenly distributed in pig brain homogenates, which were, apart from PpIX spiking, not further treated. In clinical biopsies, the microenvironment (e.g., pH) may, however, vary in local PpIX hotspots due to factors such as hypoxia.

### HI and LC–MS measurement of clinical biopsies

**Fig. 4:**
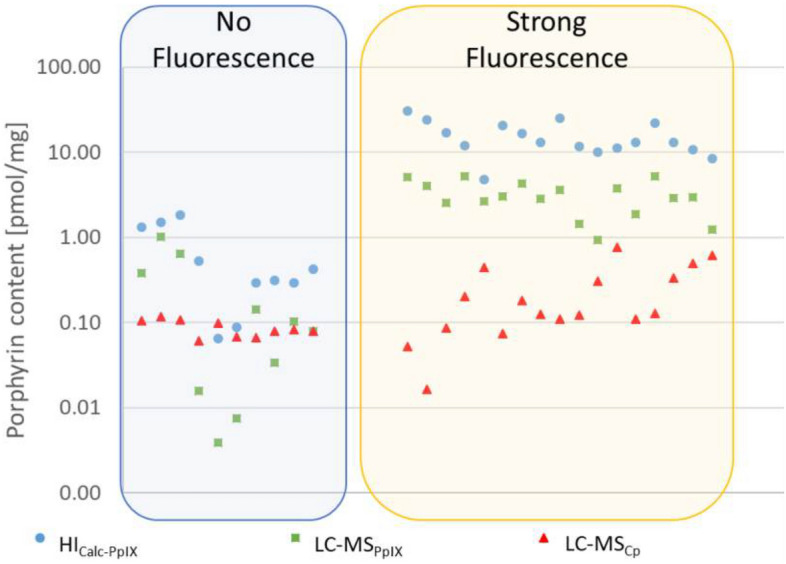
HI and LC–MS results for analyzing PpIX and Cp in clinical biopsies sorted by fluorescence grade (note logarithmic scale on the y-axis; for data, see Supplemental Digital Content 5, Table S2). Ten biopsies did not fluoresce during surgery. Blue circles: HI_Calc-PpIX_; green squares: LC-MS_PpIX_; red triangles: LC-MS_Cp_.

All patient samples were measured above the LOQ and within the established calibration range (Fig. 4; for data, see Supplemental Digital Content 9 and 10, Table S6, Fig. S4). Exemplary hyperspectral images of biopsies and their corresponding PpIX contributions are given in Supplementary Fig. S6. The Pearson correlation coefficient for PpIX values was 0.858 (*p* < 0.001), indicating a linear correlation between both techniques (for data plot, see Supplemental Digital Content 10, Fig. S4). However, the HI values for PpIX exceeded those determined by LC–MS by at least 1.5-fold, with an extreme value of 34-fold. Thereby, the incidence of deviations was more than tenfold higher for non-fluorescing biopsies (30%) than for fluorescing tissue (6%; see Supplemental Digital Content 9, Table S6) with an average of nine- and sixfold overdetermination, respectively (Table 4, see Supplemental Digital Content 12, Fig. S6). To explain this phenomenon, we need to remind the reader that the two technical approaches differ in their access to PpIX. While HI measured only the surface PpIX with a penetration depth of 100—250 µm^42–45^, for LC–MS, the entire biopsy was homogenized and extracted. Moreover, in HI, PpIX is determined pixel-by-pixel followed by averaging (for limitations and details, see reference^18^), and the HI algorithm tends to overestimate, especially at higher PpIX concentrations.

**Table 4:** Range and average porphyrin content of clinical biopsies.

	HI_PpIX_ [µg/ml]	HI_Calc-PpIX_ [pmol/mg]	LC-MS_PpIX_ [pmol/mg]	LC-MS_CP_ [pmol/mg]	HI_Calc-PpIX_/LC-MS_PpIX_
None	0.26 ± 0.26 (0.02—0.76)	0.67 ± 0.64 (0.07—1.84)	0.24 ± 0.34 (0.03—1.01)	0.09 ± 0.02 (0.06—0.12)	8.93 ± 9.94 (**1.49—33.47**)
Strong	13.55 ± 9.29 (5.02—37.43)	15.55 ± 6.82 (8.42—20.80)	3.14 ± 1.34 (0.92—5.18)	0.24 ± 0.22 (0.02—0.76)	5.51 ± 2.33 (**1.83—10.99**)

Range (in brackets) and average porphyrin content of clinical biopsies graded “none” and “strong” as determined with HI and LC–MS and their ratio. Concentrations in pmol/mg were calculated using the calibration curves obtained from pig brain experiments.

It was possible to quantify PpIX and Cp using LC–MS, even in non-fluorescing tissue. More Cp than PpIX was detected in four such biopsies, which could be attributed to a considerably lower PpIX concentration rather than an increase in Cp. However, very low concentrations at the limit of detection are always subject to measurement inaccuracies. The results confirm that PpIX is the main fluorophore determined during FGS, although the Cp concentration also increases with fluorescence intensity (Fig. 4).

## Discussion

HI is of significant interest in neurosurgery, with ongoing focus on developing it into a real-time tool for intraoperative use. With highly specific and sensitive MS-based porphyrin analysis, we assessed the PpIX values generated by HI. Both methods are very different – one measures the mass-to-charge ratio of individual ions generated from extracted molecules, and the other uses the fluorescence signal generated by in situ excitation of the molecules from the surface of a tissue sample. The latter’s advantage is that the biopsy remains intact and can be re-measured while the sample is destroyed for MS analysis. Moreover, LC–MS sample preparation and measurement take several hours, whereas HI information is provided within minutes without requiring extensive sample preparation. However, MS delivers absolute concentrations, while HI relies on a semi-specific signal and is limited by uncertainties originating from spectral overlapping of different fluorophores, varying extinction maxima, and interfering tissue optical properties.

LC–MS-based porphyrin quantification enabled the determination of Cp I/III even in small biopsies and non-fluorescing tumor tissue, which helped to assess the contribution of porphyrins other than PpIX with possible spectral overlap in HI. The contribution of Cp was about ten times lower in fluorescing biopsies than that of PpIX, so the fluorescence signal in HGG biopsies results essentially from PpIX, as generally assumed.

Calibration methods and algorithms for PpIX quantification with HI remain challenging due to the need for precise spectral resolution and accurate correction for tissue heterogeneity, light scattering, and autofluorescence effects. Our data provide a unique reference set for the camera setup and algorithm to accurately deduce the PpIX concentration from HI data, which is essential as the latter method tends to overstate it considerably. The detected overestimation of the PpIX concentration by HI is a critical finding in this study. Notably, within the lower PpIX concentration range, which is essential for discriminating tumor from healthy tissue, this systematic bias may result in incorrect delineation of tumor margins. Current HI algorithms could be refined by correction parameters obtained with a large number of comparative reference experiments on surrogate tissue. Our group has already addressed this limitation by developing a deep learning-based architecture that improves the accuracy of PpIX quantification^46^. The “true” values determined by LC–MS were compared to HI results of fluorescence phantoms and pig brain homogenates and used to determine the deviation parameter for spectral data processing^46^. This is particularly important as LC–MS is not applicable for real-time analysis.

Furthermore, the capability to differentiate reliably between porphyrin species will help to investigate the two observed PpIX photostates^22^. The joint MS-HI approach has demonstrated its potential and can be further refined by incorporating additional porphyrin species and other tumor entities.

### Limitations

Limitations of our experiments include the use of pig brain, which might differ slightly in its chemical and optical properties compared to human brain tissue. Access to healthy human brain tissue as a control is restricted to rare clinical scenarios, such as temporal lobe resection during epilepsy surgery. Additionally, in spiked pig brain surrogates, PpIX is distributed homogeneously, whereas, in the human brain, it is actively produced inhomogeneously within tumor tissue. As PpIX is influenced by its microenvironment, this might impact the fluorescing characteristics for HI measurement. Experimental determination of the properties of the in vivo PpIX microenvironment is, however, difficult, if not impossible. Moreover, this pilot study of few patients harboring a limited number of tumor entities lacks the sample numbers for far-reaching conclusions. Assessment of larger cohorts will increase the reliability of the results. Our work aims to enhance the accuracy and precision of HI in the context of developing HI instrumentation for real-time detection.

## Conclusion

HI is currently being developed for real-time analysis and future intraoperative distinction of tumor and healthy brain tissue. The use of MS-based porphyrin analysis as a reference and control method for HI detection allows to reliably determine the PpIX concentration in brain tissue using the fluorescence signal. Following the present pilot study, with a sufficiently large dataset, it is feasible to improve spectral signal processing algorithms and avoid artificial overestimations in hyperspectral assessment of 5-ALA-induced fluorescence.

## Supplementary Information

Supplementary Information.

## Data Availability

The datasets generated and/or analyzed during the current study are available in the supplementary material or from the corresponding author upon reasonable request.

## References

[1] Lacroix, M. et al. A multivariate analysis of 416 patients with glioblastoma multiforme: prognosis, extent of resection, and survival. *J. Neurosurg.* **95**, 190–198. 10.3171/jns.2001.95.2.0190 (2001). 11780887 10.3171/jns.2001.95.2.0190

[2] Stummer, W. et al. Fluorescence-guided surgery with 5-aminolevulinic acid for resection of malignant glioma: a randomised controlled multicentre phase III trial. *Lancet Oncol.* **7**, 392–401. 10.1016/S1470-2045(06)70665-9 (2006). 16648043 10.1016/S1470-2045(06)70665-9

[3] Suero Molina, E., Schipmann, S. & Stummer, W. Maximizing safe resections: the roles of 5-aminolevulinic acid and intraoperative MR imaging in glioma surgery-review of the literature. *Neurosurg. Rev.* **42**, 197–208. 10.1007/s10143-017-0907-z (2019). 28921173 10.1007/s10143-017-0907-z PMC6502775

[4] Ennis, S. R. et al. Transport of 5-aminolevulinic acid between blood and brain. *Brain Res.* **959**, 226–234. 10.1016/S0006-8993(02)03749-6 (2003). 12493610 10.1016/s0006-8993(02)03749-6

[5] Colditz, M. J., van Leyen, K. & Jeffree, R. L. Aminolevulinic acid (ALA)-protoporphyrin IX fluorescence guided tumour resection. Part 2: theoretical, biochemical and practical aspects. *J. Clin. Neurosci. Off. J. Neurosurg. Soc. Australasia* **19**, 1611–1616. 10.1016/j.jocn.2012.03.013 (2012). 10.1016/j.jocn.2012.03.013 23059058

[6] McNicholas, K., MacGregor, M. N. & Gleadle, J. M. In order for the light to shine so brightly, the darkness must be present-why do cancers fluoresce with 5-aminolaevulinic acid?. *Br. J. Cancer* **121**, 631–639. 10.1038/s41416-019-0516-4 (2019). 31406300 10.1038/s41416-019-0516-4 PMC6889380

[7] Kiening, M. & Lange, N. A recap of heme metabolism towards understanding protoporphyrin IX selectivity in cancer cells. *Int. J. Mol. Sci.*10.3390/ijms23147974 (2022). 35887311 10.3390/ijms23147974 PMC9324066

[8] Shemin, D. & Rittenberg, D. The biological utilization of glycine for the synthesis of the protoporphyrin of hemoglobin. *J. Biol. Chem.* **166**, 621–625. 10.1016/S0021-9258(17)35200-6 (1946). 20276176

[9] Sachar, M., Anderson, K. E. & Ma, X. Protoporphyrin IX: the good, the bad, and the ugly. *J. Pharmacol. Exp. Ther.* **356**, 267–275. 10.1124/jpet.115.228130 (2016). 26588930 10.1124/jpet.115.228130 PMC4727154

[10] Walke, A., Suero Molina, E., Stummer, W. & König, S. Protoporphyrin IX analysis from blood and serum in the context of neurosurgery of glioblastoma. In *Mass Spectrometry in Life Sciences and Clinical Laboratory* (ed. Mitulović, G.) (IntechOpen, 2021).

[11] Heinemann, I. U., Jahn, M. & Jahn, D. The biochemistry of heme biosynthesis. *Arch. Biochem. Biophys.* **474**, 238–251. 10.1016/j.abb.2008.02.015 (2008). 18314007 10.1016/j.abb.2008.02.015

[12] Beika, M. et al. Accumulation of uroporphyrin i in necrotic tissues of squamous cell carcinoma after administration of 5-aminolevulinic acid. *Int. J. Mol. Sci.*10.3390/ijms221810121 (2021). 34576284 10.3390/ijms221810121 PMC8471361

[13] Stummer, W. et al. Technical principles for protoporphyrin-IX-fluorescence guided microsurgical resection of malignant glioma tissue. *Acta Neurochir.* **140**, 995–1000. 10.1007/s007010050206 (1998). 9856241 10.1007/s007010050206

[14] Johansson, A. et al. 5-aminolevulinic acid-induced protoporphyrin IX levels in tissue of human malignant brain tumors. *Phys. Rev. E*10.1103/PhysRevE.102.042905 (2020). 10.1111/j.1751-1097.2010.00799.x 20854414

[15] Yoneda, T. et al. Spectral radiance of protoporphyrin ix fluorescence and its histopathological implications in 5-aminolevulinic acid-guided surgery for glioblastoma. *Photomed. Laser Surg.* **36**, 266–272. 10.1089/pho.2017.4384 (2018). 29480754 10.1089/pho.2017.4384

[16] Suero Molina, E., Stögbauer, L., Jeibmann, A., Warneke, N. & Stummer, W. Validating a new generation filter system for visualizing 5-ALA-induced PpIX fluorescence in malignant glioma surgery: a proof of principle study. *Acta Neurochir.* **162**, 785–793. 10.1007/s00701-020-04227-7 (2020). 32034493 10.1007/s00701-020-04227-7 PMC7066295

[17] Petrecca, K., Guiot, M. C., Panet-Raymond, V. & Souhami, L. Failure pattern following complete resection plus radiotherapy and temozolomide is at the resection margin in patients with glioblastoma. *J. Neurooncol.* **111**, 19–23. 10.1007/s11060-012-0983-4 (2013). 23054563 10.1007/s11060-012-0983-4

[18] Walke, A. et al. Challenges in, and recommendations for, hyperspectral imaging in ex vivo malignant glioma biopsy measurements. *Sci. Rep.* **13**, 3829. 10.1038/s41598-023-30680-2 (2023). 36882505 10.1038/s41598-023-30680-2 PMC9992662

[19] Valdés, P. A. et al. Quantitative, spectrally-resolved intraoperative fluorescence imaging. *Sci. Rep.* **2**, 798. 10.1038/srep00798 (2012). 23152935 10.1038/srep00798 PMC3497712

[20] Li, Q. et al. Review of spectral imaging technology in biomedical engineering: achievements and challenges. *J. Biomed. Opt.* **18**, 100901. 10.1117/1.JBO.18.10.100901 (2013). 24114019 10.1117/1.JBO.18.10.100901

[21] Valdés, P. A. et al. A spectrally constrained dual-band normalization technique for protoporphyrin IX quantification in fluorescence-guided surgery. *Opt. Lett.* **37**, 1817–1819. 10.1364/OL.37.001817 (2012). 22660039 10.1364/OL.37.001817 PMC3774026

[22] Black, D. et al. Characterization of autofluorescence and quantitative protoporphyrin IX biomarkers for optical spectroscopy-guided glioma surgery. *Sci. Rep.* **11**, 20009. 10.1038/s41598-021-99228-6 (2021). 34625597 10.1038/s41598-021-99228-6 PMC8501114

[23] Black, D., Liquet, B., Di Ieva, A., Stummer, W. & Suero Molina, E. Spectral library and method for sparse unmixing of hyperspectral images in fluorescence guided resection of brain tumors. *Biomed. Opt. Express* **15**, 4406–4424. 10.1364/BOE.528535 (2024). 39346979 10.1364/BOE.528535 PMC11427211

[24] Alston, L., Rousseau, D., Hebert, M., Mahieu-Williame, L. & Montcel, B. Nonlinear relation between concentration and fluorescence emission of protoporphyrin IX in calibrated phantoms. *J. Biomed. Opt.* **23**, 1–7. 10.1117/1.JBO.23.9.097002 (2018). 30251489 10.1117/1.JBO.23.9.097002

[25] Suero Molina, E. et al. Unraveling the blue shift in porphyrin fluorescence in glioma: The 620 nm peak and its potential significance in tumor biology. *Front. Neurosci.* **17**, 1261679. 10.3389/fnins.2023.1261679 (2023). 38027504 10.3389/fnins.2023.1261679 PMC10657867

[26] Scolaro, L. M. et al. Aggregation behavior of protoporphyrin IX in aqueous solutions: clear evidence of vesicle formation. *J. Phys. Chem. B* **106**, 2453–2459. 10.1021/jp013155h (2002).

[27] Melø, T. B. & Reisaeter, G. The physicochemical state of protoporphyrin IX in aqueous solution investigated by fluorescence and light scattering. *Biophys. Chem.* **25**, 99–104. 10.1016/0301-4622(86)85070-0 (1986). 17010276 10.1016/0301-4622(86)85070-0

[28] Seo, I., Tseng, S. H., Cula, G. O., Bargo, P. R. & Kollias, N. Fluorescence spectroscopy for endogenous porphyrins in human facial skin. *Photon. Ther. Diagn. V*10.1117/12.811913 (2009).

[29] Dietel, W., Pottier, R., Pfister, W., Schleier, P. & Zinner, K. 5-Aminolaevulinic acid (ALA) induced formation of different fluorescent porphyrins: a study of the biosynthesis of porphyrins by bacteria of the human digestive tract. *J. Photochem. Photobiol. B Biol.* **86**, 77–86. 10.1016/j.jphotobiol.2006.07.006 (2007). 10.1016/j.jphotobiol.2006.07.006 16973372

[30] Lennon, Á. M., Brune, L., Techert, S. & Buchalla, W. Fluorescence spectroscopy shows porphyrins produced by cultured oral bacteria differ depending on composition of growth media. *Caries Res.* **57**, 74–86. 10.1159/000528731 (2023). 36529123 10.1159/000528731 PMC10137314

[31] Dysart, J. S. & Patterson, M. S. Photobleaching kinetics, photoproduct formation, and dose estimation during ALA induced PpIX PDT of MLL cells under well oxygenated and hypoxic conditions. *Photochem. Photobiol. Sci. Off. J. Eur. Photochem. Assoc. Eur. Soc. Photobiol.* **5**, 73–81. 10.1039/B511807G (2006). 10.1039/b511807g 16395430

[32] Juzenas, P., Iani, V., Bagdonas, S., Rotomskis, R. & Moan, J. Fluorescence spectroscopy of normal mouse skin exposed to 5-aminolaevulinic acid and red light. *J. Photochem. Photobiol. B Biol.* **61**, 78–86. 10.1016/S1011-1344(01)00149-X (2001). 10.1016/s1011-1344(01)00149-x 11485851

[33] Kim, A., Khurana, M., Moriyama, Y. & Wilson, B. C. Quantification of in vivo fluorescence decoupled from the effects of tissue optical properties using fiber-optic spectroscopy measurements. *J. Biomed. Opt.* **15**, 67006. 10.1117/1.3523616 (2010). 10.1117/1.3523616 PMC3025598 21198210

[34] Valdés, P. A. et al. Quantitative fluorescence in intracranial tumor: implications for ALA-induced PpIX as an intraoperative biomarker. *J. Neurosurg.* **115**, 11–17. 10.3171/2011.2.JNS101451 (2011). 21438658 10.3171/2011.2.JNS101451 PMC3129387

[35] Walke, A., Krone, C., Stummer, W., König, S. & Suero Molina, E. Protoporphyrin IX in serum of high-grade glioma patients: A novel target for disease monitoring via liquid biopsy. *Sci. Rep.* **14**, 4297 (2024). 38383693 10.1038/s41598-024-54478-y PMC10881484

[36] Adams, K. J. et al. Skyline for small molecules: a unifying software package for quantitative metabolomics. *J. Proteome Res.* **19**, 1447–1458. 10.1021/acs.jproteome.9b00640 (2020). 31984744 10.1021/acs.jproteome.9b00640 PMC7127945

[37] Kessner, D., Chambers, M., Burke, R., Agus, D. & Mallick, P. ProteoWizard: open source software for rapid proteomics tools development. *Bioinformatics (Oxford England)* **24**, 2534–2536. 10.1093/bioinformatics/btn323 (2008). 18606607 10.1093/bioinformatics/btn323 PMC2732273

[38] Sullivan, S. A. et al. Mass-spectrometric profiling of porphyrins in complex biological samples with fundamental, toxicological, and pharmacological applications. *Anal. Biochem.* **478**, 82–89. 10.1016/j.ab.2015.03.004 (2015). 25769421 10.1016/j.ab.2015.03.004 PMC4410076

[39] Louis, D. N. et al. The 2021 WHO classification of tumors of the central nervous system: a summary. *Neuro Oncol.* **23**, 1231–1251. 10.1093/neuonc/noab106 (2021). 34185076 10.1093/neuonc/noab106 PMC8328013

[40] Danton, M. & Lim, C. K. Porphyrin profiles in blood, urine and faeces by HPLC/electrospray ionization tandem mass spectrometry. *Biomed. Chromatogr. BMC* **20**, 612–621. 10.1002/bmc.656 (2006). 16779779 10.1002/bmc.656

[41] Fyrestam, J., Bjurshammar, N., Paulsson, E., Johannsen, A. & Östman, C. Determination of porphyrins in oral bacteria by liquid chromatography electrospray ionization tandem mass spectrometry. *Anal. Bioanal. Chem.* **407**, 7013–7023. 10.1007/s00216-015-8864-2 (2015). 26168965 10.1007/s00216-015-8864-2 PMC4551553

[42] Valdes, P. A., Juvekar, P., Agar, N. Y. R., Gioux, S. & Golby, A. J. Quantitative wide-field imaging techniques for fluorescence guided neurosurgery. *Front. Surg.* **6**, 31. 10.3389/fsurg.2019.00031 (2019). 31245380 10.3389/fsurg.2019.00031 PMC6563771

[43] Wei, L., Roberts, D. W., Sanai, N. & Liu, J. T. C. Visualization technologies for 5-ALA-based fluorescence-guided surgeries. *J. Neurooncol.* **141**, 495–505. 10.1007/s11060-018-03077-9 (2019). 30554344 10.1007/s11060-018-03077-9 PMC7707111

[44] Konecky, S. D. et al. Spatial frequency domain tomography of protoporphyrin IX fluorescence in preclinical glioma models. *J. Biomed. Opt.* **17**, 56008 (2012). 10.1117/1.JBO.17.5.056008 PMC3381025 22612131

[45] Roberts, D. W. et al. Red-light excitation of protoporphyrin IX fluorescence for subsurface tumor detection. *J. Neurosurg.* **128**, 1690–1697. 10.3171/2017.1.JNS162061 (2018). 28777025 10.3171/2017.1.JNS162061 PMC5797501

[46] Black, D. et al. Deep learning-based correction and unmixing of hyperspectral images for brain tumor surgery. *iScience* **27**(12), 111273 (2024). 39628576 10.1016/j.isci.2024.111273 PMC11613202

