# Supplementary material for: Integrating mass spectrometry and hyperspectral imaging for protoporphyrin IX detection in malignant glioma tissue
Source: Sci Rep. 2025 Nov 4;15:38460. doi: 10.1038/s41598-025-26245-0 (PMC12586713; doi:10.1038/s41598-025-26245-0)
Supplement: Supplementary file 1 — Supplementary Information. [file 41598_2025_26245_MOESM1_ESM.pdf]

Table S1: *Instrument parameters for LC-MS experiments using an Esquire3000 ion trap coupled to an analytical HPLC (HP1100).*

**LC Parameter**		
**Column**	Poroshell C_{18} (2.7 µm, 2.1 mm i.d., 100 mm) with guard column	
**Flow**	0.3 ml/min	
**Solvent A**	94% H_2O/ 5% ACN / 1% FA	
**Solvent B**	94% ACN / 5% H_2O / 1% FA	
**Gradient**	t [min]	Solvent B [%]
	0	20
	3	35
	9	35
	9.5	100
	15.5	100
	16	20
	20	20
**Injection volume**	10 µl	
**MS Parameter**		
**Source**	ESI +	
**Capillary**	-4,500 V	
**End plate**	-500 V	
**Nebulizer**	30.0 psi	
**Dry gas**	7.0 l/min	
**Dry temperature**	300 °C	
**Scan range**	*m*/*z* 400-850	

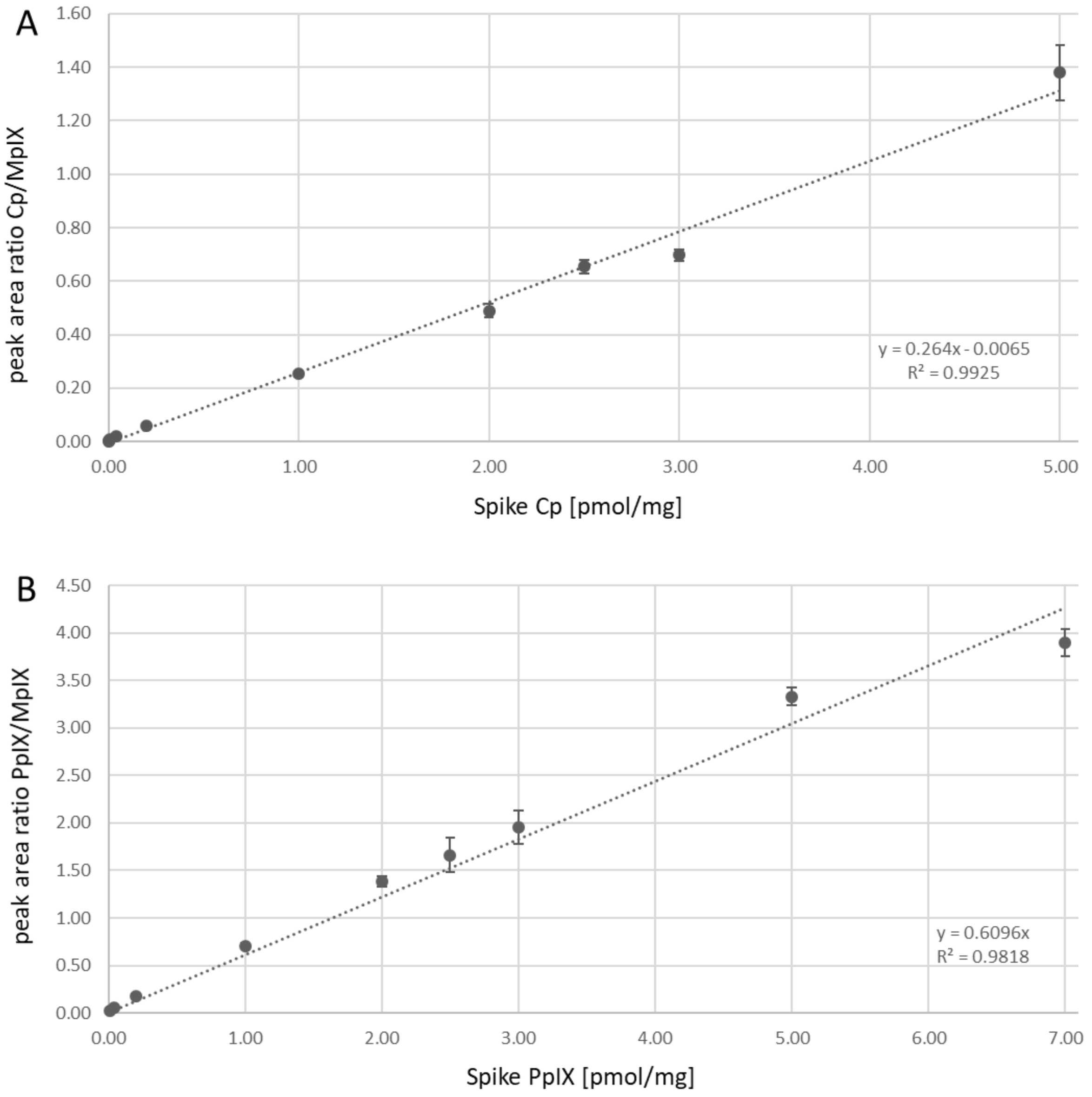

Figure S1: *LC-MS peak area ratios of spiked pig brain homogenates: A) total Cp /MpIX (Cp I / III denoted as total Cp), and B) PpIX/MpIX. The spiked porphyrin concentration is plotted against the peak area ratio of the LC-MS signal of the analyte and MpIX as the internal standard as determined with Skyline. MpIX was co-run as internal standard.*

Table S2: *Recovery rates were determined by comparing MS results of spiked pig brain homogenate with PpIX, Cp I and Cp III (each at a concentration of 1pmol/mg). Samples A & B were spiked prior to the extraction process, whereas for C the porphyrins were added after the extraction and reconstitution in DMSO; Avg = average; SD = standard deviation; Var = variation. The experiment was performed with 3 technical replicates.*

**Sample**	**Spike**	**Avg FA Total CP /ISTD**	**SD**	**Var**	**Avg FA PpIX /ISTD**	**SD2**	**Var 2**	**Recovery rate Total CP**	**SD3**	**Var 3**	**Recovery rate PpIX**	**SD4**	**Var 4**
**A**	into pig brain	0.39	0.01	0.0219	0.90	0.02	0.0220	**0.48**	0.00	0.0074	**0.79**	0.00	0.0062
**B**	into pig brain	0.37	0.01	0.0327	0.95	0.08	0.0813	**0.43**	0.01	0.0175	**0.81**	0.04	0.0443
**C**	after reconstitution in DMSO	0.78	0.02	0.0232	1.09	0.01	0.0110	1.00	0.01	0.0085	1.00	0.04	0.0200

Table S3: *Matrix effects were determined by comparing MS peak areas of a control, which consisted of DMSO and the spiked porphyrins, with an extract of pig brain homogenate, where porphyrins were added after reconstitution in DMSO.*

**Sample**	**CpI**	**SD**	**Var**	**CpIII**	**SD2**	**Var2**
**Matrix**	1,284,674.33	19,840.27	0.0154	2,274,127.33	19,511.02	0.0086
**Control**	1,249,275.83	44,399.29	0.0355	2,272,734.33	84,979.25	0.0374
**Matrix effect**	**1.03**			**1.0**		
**Sample**	**MPIX**	**SD3**	**Var 3**	**PPIX**	**SD4**	**Var 4**
**Matrix**	4,656,734.333	76,719.02	0.0165	5,056,010.33	101,163.47	0.0200
**Control**	4,930,994	163,943.73	0.0332	2,561,282.83	155,384.67	0.0607
**Matrix effect**	**0.94**			**1.97**		

Table S4: *For LC-MS, precision was determined by measuring the peak area ratio of three identical samples and analyzing the degree of random variation. Within the tested range, precision ranged from 5-14%, except for the lowest concentration, which had a precision of 30% for PpIX and 3-11% for Cp.*

**PpIX Concentration [pmol/mg]**	**Average PpIX /ISTD**	**SD**	**Var**
**0**	0	0.01	**0.07**
**0.01**	0.0231	0.01	**0.32**
**0.04**	0.0624	0.01	**0.10**
**0.2**	0.1820	0.02	**0.14**
**1**	0.7081	0.06	**0.08**
**2**	1.3819	0.18	**0.13**
**2.5**	1.6625	0.17	**0.10**
**3**	1.9550	0.09	**0.05**
**Cp Concentration [pmol/mg]**	**Average peak area ratio Cp/ISTD**	**SD2**	**Var2**
**0**	0.0023	0.0002	**0.10**
**0.02**	0.0074	0.0003	**0.04**
**0.08**	0.0207	0.0022	**0.11**
**0.4**	0.0586	0.0030	**0.05**
**2**	0.2527	0.0098	**0.04**
**4**	0.4896	0.0237	**0.05**
**5**	0.6538	0.0250	**0.04**
**6**	0.6973	0.0204	**0,03**

Table S5: *Measurement accuracy for PpIX detection was tested in pig brain homogenate by spiking three samples with a known concentration of 3.0 pmol/mg PpIX, and subsequently using the developed protocol for PpIX quantification. Thereby, an accuracy of 100.2 ± 6% was achieved.*

**Sample**	**Spiked PpIX [pmol/mg]**	**$LC\text{-}MS_{PpIX}$ [pmol/mg]**	**PpIX Accuracy [%]**	**Average**	**SD**	**Var**
**A**	3	2.94	94.77	**100.16**	**6.09**	**0.06**
**B**	3	3.07	98.94			
**C**	3	3.30	106.76			

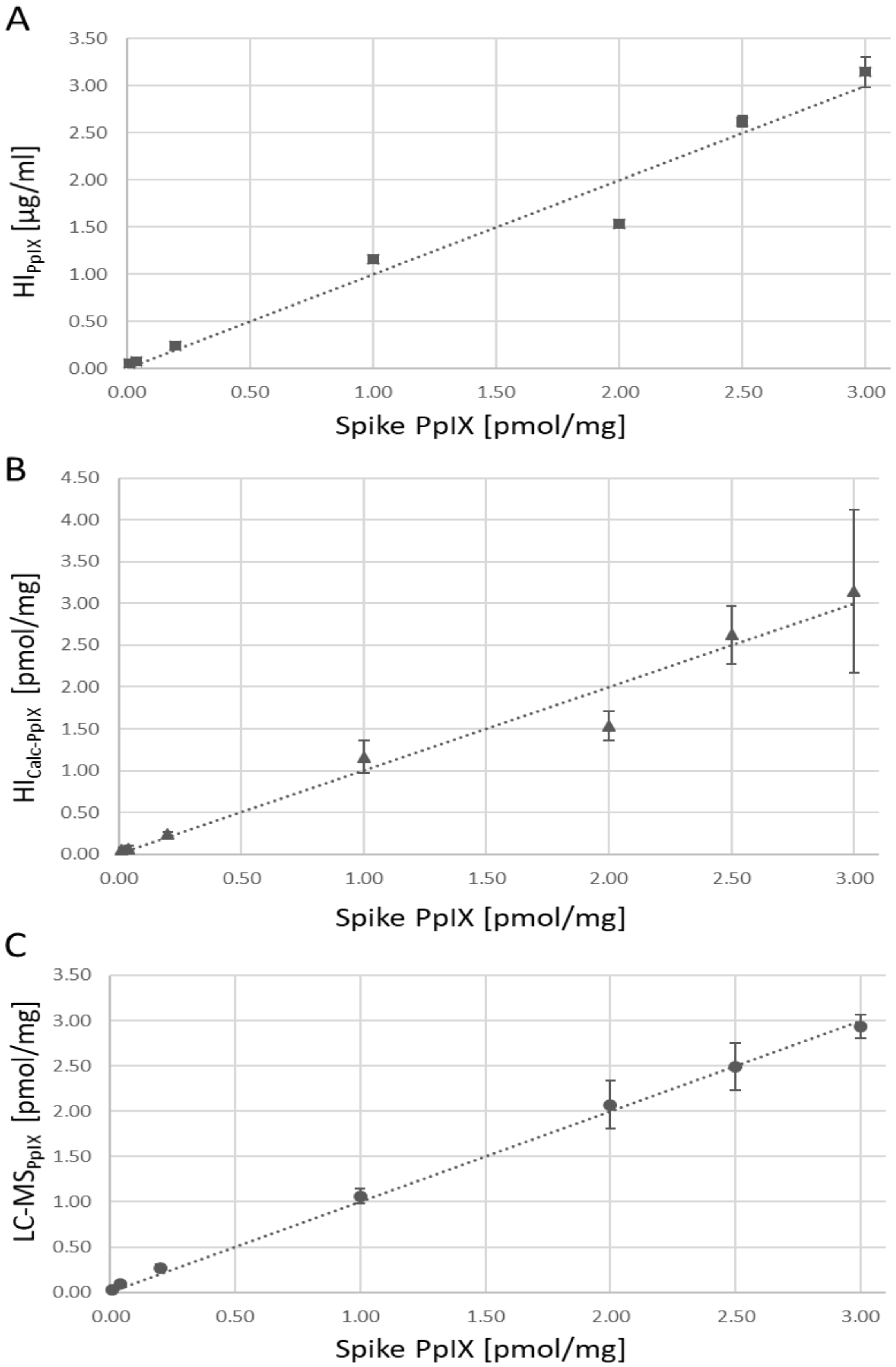

Figure S2: *PpIX measurements of spiked pig brain homogenate with* ***A/B)*** *HI and* ***C)*** *LC-MS.*

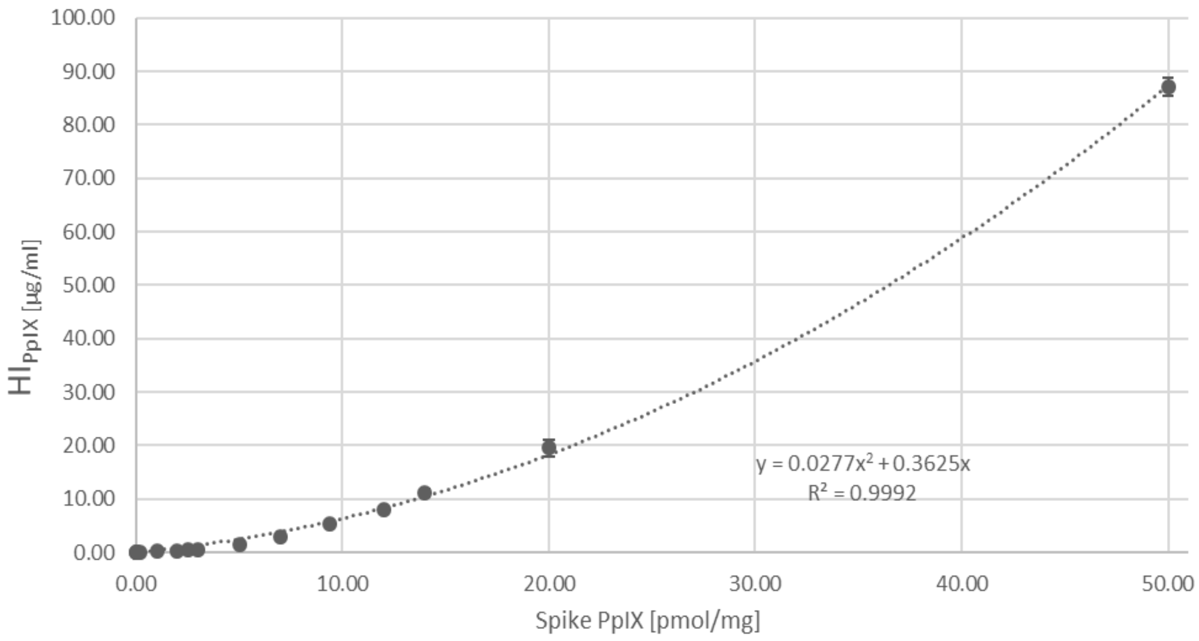

Figure S3: *HI measurement results of spiked pig brain homogenates for concentrations up to 50 pmol/mg.*

Table S6: *Porphyrin concentration in patients' biopsies as measured with HI and LC-MS sorted by fluorescence grade. Cp was determined only with LC-MS. The PpIX concentration considerably exceeded that of Cp in most cases of visible fluorescence. Biopsies labeled in red were categorized as fluorescing during surgery whereas all others showed no fluorescence.*

**Serial number**	**$HI_{Calc-PpIX}$ [pmol/mg]**	**$LC\text{-}MS_{PpIX}$ [pmol/mg]**	**$LC\text{-}MS_{Cp}$ [pmol/mg]**	**Fluorescence (1=strong; 0=none)**	**Ratio $HI_{Calc-PpIX}$/ $LC\text{-}MS_{PpIX}$**
1	1.32	0.38	0.11	0	3.5
2	1.50	1.01	0.12	0	1.5
3	1.84	0.65	0.11	0	2.9
4	0.52	0.02	0.06	0	33.5
5	0.07	0.00	0.10	0	16.7
6	0.09	0.01	0.07	0	11.8
7	0.29	0.14	0.07	0	2.0
8	0.31	0.03	0.08	0	9.3
9	0.29	0.10	0.08	0	2.8
10	0.42	0.08	0.08	0	5.4
11	30.80	5.10	0.05	1	6.0
12	23.81	4.02	0.02	1	5.9
13	16.93	2.54	0.09	1	6.7
14	12.06	5.17	0.20	1	2.3
15	4.83	2.63	0.45	1	1.8
16	20.49	3.03	0.07	1	6.8
17	16.72	4.28	0.18	1	3.9
18	13.10	2.83	0.13	1	4.6
19	25.19	3.60	0.11	1	7.0
20	11.83	1.42	0.12	1	8.3
21	10.11	0.92	0.31	1	11.0
22	11.31	3.77	0.76	1	3.0
23	13.07	1.87	0.11	1	7.0
24	21.97	5.18	0.13	1	4.2
25	13.17	2.89	0.33	1	4.6
26	10.62	2.93	0.49	1	3.6
27	8.42	1.24	0.61	1	6.8

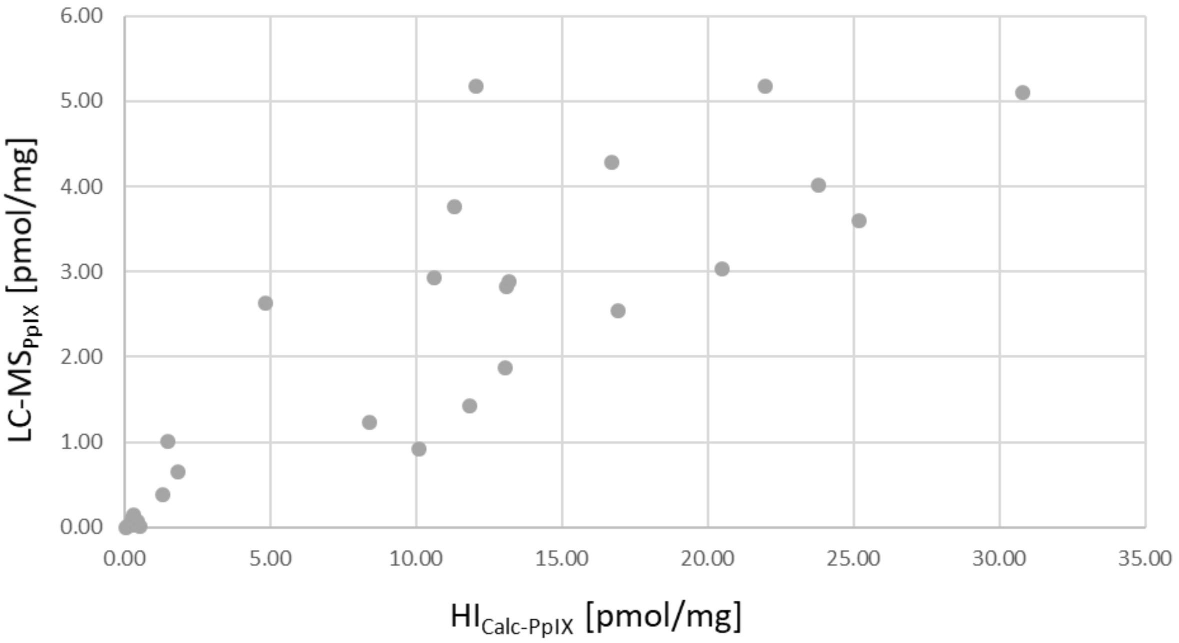

Figure S4: *Correlation of HI and LC-MS measurements of clinical biopsies.*

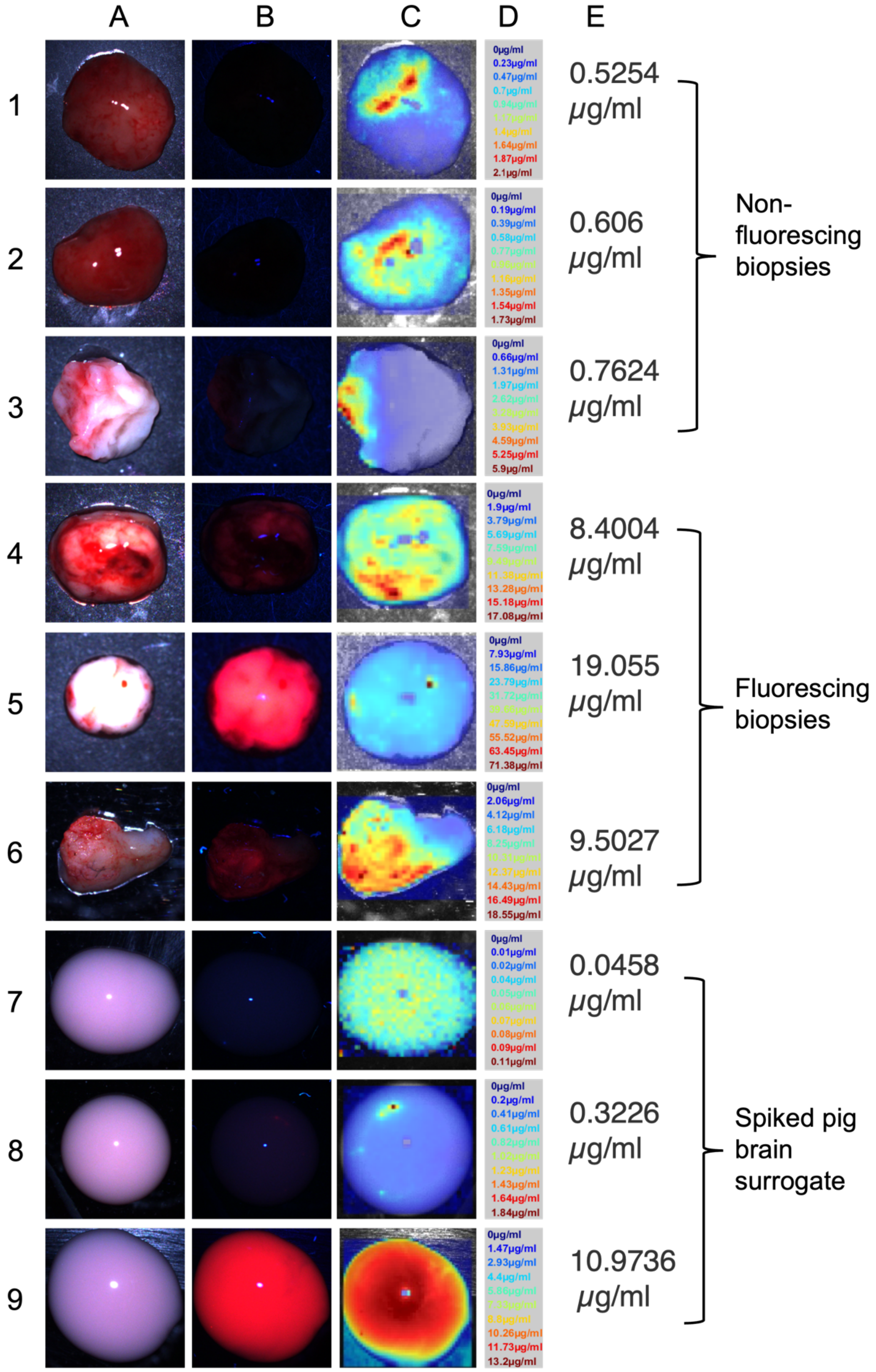

Figure S5: *Representative set of hyperspectral images of biopsies and pig brain surrogate. (A) Color image; (B) BLUE 400; (C) Hyperspectral overlay; (D) PpIX contribution [µg/ml] per pixel; (E) Average PpIX contribution [µg/ml]. (1) Biopsy 1; (2) Biopsy 2; (3) Biopsy 3; (4) Biopsy 14; (5) Biopsy 16; (6) Biopsy 19; (7) Pig brain surrogate spiked with 0.001 pmol/mg PpIX; (8) Pig brain surrogate spiked with 2.00 pmol/mg PpIX; (9) Pig brain surrogate spiked with 14.0 pmol/mg PpIX.*

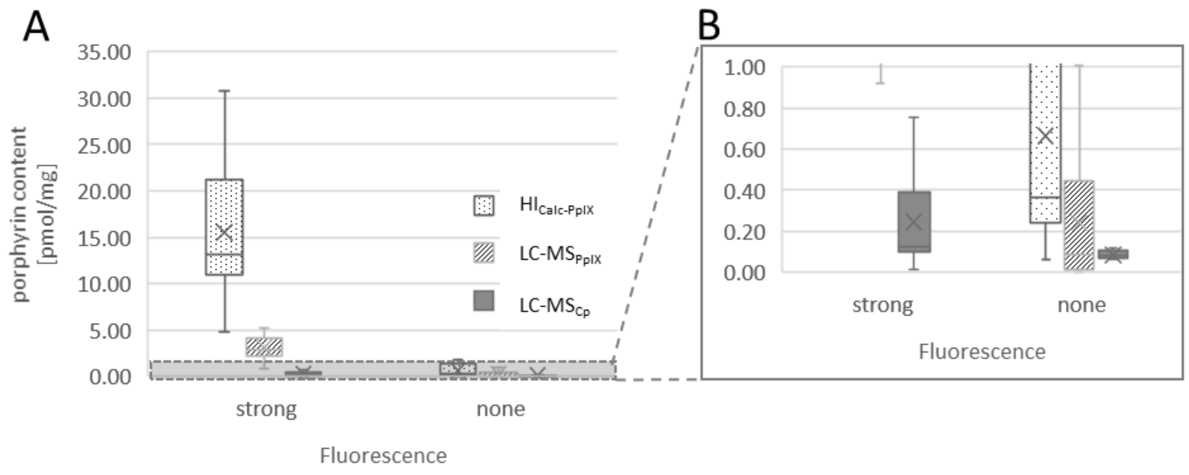

Figure S6: *Porphyrin content in tissue with respect to fluorescence quality as rated by the surgeon (none, strong). A) PpIX as determined by HI (dotted), PpIX (striped) and Cp (solid) as determined by LC-MS. B) Zoom into the lower range of up to 1 pmol/mg.*
